# Temporal increase in HIV-1 non-R5 tropism frequency among antiretroviral-naive patients from northern Poland

**DOI:** 10.7448/IAS.17.4.19687

**Published:** 2014-11-02

**Authors:** Milosz Parczewski, Magdalena Leszczyszyn-Pynka, Magdalena Witak-Jêdra, Katarzyna Maciejewska, Anna Urbañska

**Affiliations:** Department of Infectious, Tropical Diseases, Pomeranian Medical University in Szczecin, Szczecin, Poland

## Abstract

**Introduction:**

Sequencing of the third hypervariable loop allows to identify genotype-based HIV tropism. R5-tropic viruses associated with early stages of infection are preferentially transmitted, while non-R5 HIV-1 tropism has been associated with severe immunodeficiency and lower lymphocyte CD4 nadir and may reflect delayed HIV diagnosis. In this study, we investigate the changes in tropism frequency from 2007 to 2013.

**Materials and Methods:**

Study included 194 patients with confirmed HIV infection linked to care in 2007–2013. Baseline plasma samples from treatment naive patients were used for HIV-1 genotypic tropism assessment based on triplicate V3 loop sequencing. Non-R5 tropism prediction thresholds were assigned using a false positive rate (FPR) of 10% and 5.75% FPR and associated with clinical and laboratory data (age, gender, date of HIV diagnosis, route of transmission, CDC clinical category at diagnosis, pretreatment HIV viral load, baseline and nadir lymphocyte CD4 counts). For statistics, chi-square and Mann–Whitney U tests were used, time trends were examined using logistic regression (R statistical platform, v. 3.1.0) for binary variables and linear regression for continuous ones.

**Results:**

Overall non-R5 tropism frequency for the 5.75% FPR was 15.5% and 27.8% for 10% FPR. Frequency of the non-R5 tropism predicted using 5.75% FPR increased significantly from 2007 (0%) to 2013 (25%) [OR: 1.44 (95% CI 1.14–1.86), p=0.003, rough slope +3.89%/year] (Figure 1a). With 10% FPR, the frequency changed from 7% (2007) to 33% (2013) [OR: 1.17 (95% CI 0.99–1.39), p=0.054, rough slope +3.0%/year] (Figure 1b). Baseline lymphocyte CD4 count and nadir, as well as pretreatment HIV-1 viral loads were stable over time of observation (r=0.014, p=0.84; r=0.13, p=0.085; r=0.016, p=0.83 for CD4 baseline, nadir and HIV load, respectively). Frequency of AIDS at HIV diagnosis increased from 21.4% in 2007 to 38.0% in 2013, however trend over time was insignificant [OR: 1.1 (95% CI 0.95–1.31), p=0.19]. Temporal trends for HIV transmission route, gender, non-B variant frequencies also were not significant.

**Conclusions:**

R5 tropism predominates among the treatment naive individuals but increase in the frequency of non-R5 tropic variants may limit clinical efficacy of the coreceptor inhibitors. Increased prevalence of non-R5 HIV-1 may be related to late care entry and higher number of AIDS diagnoses in the recent years.

**Figure 1:**
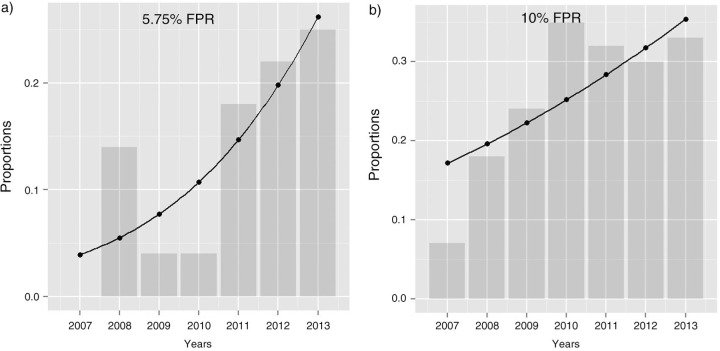
Logistic regression analysis of the V3 loop genotype-based HIV-1 tropism frequency: (a) tropism frequencies with PFR 5.75%; (b) tropism frequencies with 10% PFR. Observed tropism frequencies are shown as bars with superimposed logistic regression curve.

